# α-Synuclein enhances histone H3 lysine-9 dimethylation and H3K9me2-dependent transcriptional responses

**DOI:** 10.1038/srep36328

**Published:** 2016-11-03

**Authors:** Naoto Sugeno, Sandra Jäckel, Aaron Voigt, Zinah Wassouf, Julia Schulze-Hentrich, Philipp J. Kahle

**Affiliations:** 1 Laboratory of Functional Neurogenetics, Department of Neurodegeneration, Hertie Institute for Clinical Brain Research, Faculty of Medicine, University of Tübingen, 72076 Tübingen, Germany; 2 Department of Neurology, Tohoku University Graduate School of Medicine, Sendai, Miyagi 980-8574, Japan; 3 German Center for Neurodegenerative Diseases, 72076 Tübingen, Germany; 4 Department of Neurology, RWTH University Aachen, 52074 Aachen, Germany; 5 JARA-Institute Molecular Neuroscience and Neuroimaging, Forschungszentrum Jülich GmbH and RWTH Aachen University, 52074 Aachen, Germany; 6 Epigenetics Group, Institute of Medical Genetics and Applied Genomics, Faculty of Medicine, University of Tübingen, 72076 Tübingen, Germany

## Abstract

α-Synuclein (αS) is a protein linked to Parkinson’s disease (PD) and related neurodegenerative disorders. It is mostly localized within synapses, but αS has also been suggested to play a role in the nucleus. We used transgenic *Drosophila* and inducible SH-SY5Y neuroblastoma cells to investigate the effects of αS on chromatin with a particular focus on histone modifications. Overexpression of αS in male flies as well as in retinoic acid pre-treated neuroblastoma cells led to an elevation of histone H3K9 methylations, mostly mono- (H3K9me1) and di- (H3K9me2). The transient increase of H3K9 methylation in αS-induced SH-SY5Y cells was preceded by mRNA induction of the euchromatic histone lysine *N*-methyltransferase 2 (*EHMT2*). EHMT2 and H3K9me2 can function within the REST complex. Chromatin immunoprecipitation (ChIP) analyses of selected candidate, REST regulated genes showed significantly increased H3K9me2 promoter occupancy of genes encoding the L1CAM cell adhesion molecule and the synaptosomal-associated protein SNAP25, whose reduced expression levels were confirmed by RT-qPCR in αS induced cells. Treatment with EHMT inhibitor UNC0638 restored the mRNA levels of *L1CAM* and *SNAP25*. Thus, αS overexpression enhances H3K9 methylations via ΕΗΜΤ2 resulting in elevated H3K9me2 at the *SNAP25* promoter, possibly affecting SNARE complex assembly and hence synaptic vesicle fusion events regulated by αS.

Parkinson’s disease (PD) is the second most prevalent neurodegenerative disease. The pathological hallmarks of PD and related disorders are proteinaceous aggregates mainly composed of α-synuclein (αS)^1^. Rare missense mutations in the αS gene (*SNCA*) cause PD^2^ with high penetrance, but multiplications of the *SNCA* locus also trigger PD syndromes in a gene dose-dependent manner, indicating that deregulated wild-type (wt) αS can also cause neurodegenerative disease^3^. Moreover, *SNCA* is a major genetic risk factor for PD, as determined in genome-wide association studies^4^,^5^. There are many leads what factors drive α-synucleinopathy^6^, including epigenetic mechanisms.

The first synuclein was identified in 1988 from *Torpedo californica* as a protein that localized to presynaptic nerve terminals and nuclei of neurons^7^. Presynaptic αS modulates the cycling of synaptic vesicles^8^, whereas roles of nuclear αS remain to be established. Proper gene expression is crucial for the cell, and therefore it is tightly regulated by the binding of regulatory proteins to promoter regions and by epigenetic alterations of chromatin structure i.e. by DNA methylation and histone modifications^9^. αS might have a potential to affect epigenetic events because it may be found in the nucleus where it can bind to histones^10^. Indeed, αS has been reported to alter histone acetylation status^11^,^12^.

Histone H3 di- or tri-methylated at lysine-9 (H3K9me2 and H3K9me3, respectively) is well characterized in heterochromatic regions, where it is implicated in repressed gene transcription^13^. Heterochromatin is categorized into two groups, constitutive heterochromatin and facultative heterochromatin^9^. Genes within constitutive heterochromatin are conventionally silent whereas the facultative heterochromatin allows genes to resume a transcriptionally active state^14^. Although it is difficult to distinguish completely, H3K9me3 is relatively abundant in constitutive heterochromatin, whereas H3K9me2 is found in facultative heterochromatin. Indeed, H3K9me2 is implicated with memory consolidation^15^ and cocaine-induced neuronal plasticity^16^. Formation of the H3K9me2 mark can be catalyzed by euchromatic histone-lysine *N*-methyltransferase 2, EHMT2 (also known as G9a, KMT1C)^17^. Enzymatic end products by EHMT2 are limited to mono- and di-methylated H3K9 through an evolutionarily conserved Su(var)3–9, E(z) and trithorax (SET) domain^18^. EHMT2/G9a is a multipotent enzyme whose functions may not be limited to H3K9 methylation^18^. Also, it is not completely understood how EHMT2 is targeted to specific chromatin positions, but some interacting transcription factors are known. Repressor element-1 (RE1)-silencing transcription factor (REST, also known as neuron-restrictive silencer factor, NRSF) can interact with EHMT2^19^. REST binds to RE1 and subsequently inactivates transcription through EHMT2-mediated H3K9 methylation^20^.

In this study, we examined global histone modifications upon αS overexpression in transgenic *Drosophila* and in inducible human neuroblastoma SH-SY5Y cells, and explored epigenetic effects and functional alterations initiated by αS.

## Results

### Enhanced H3K9 methylations in αS transgenic flies

**Figure 1:**
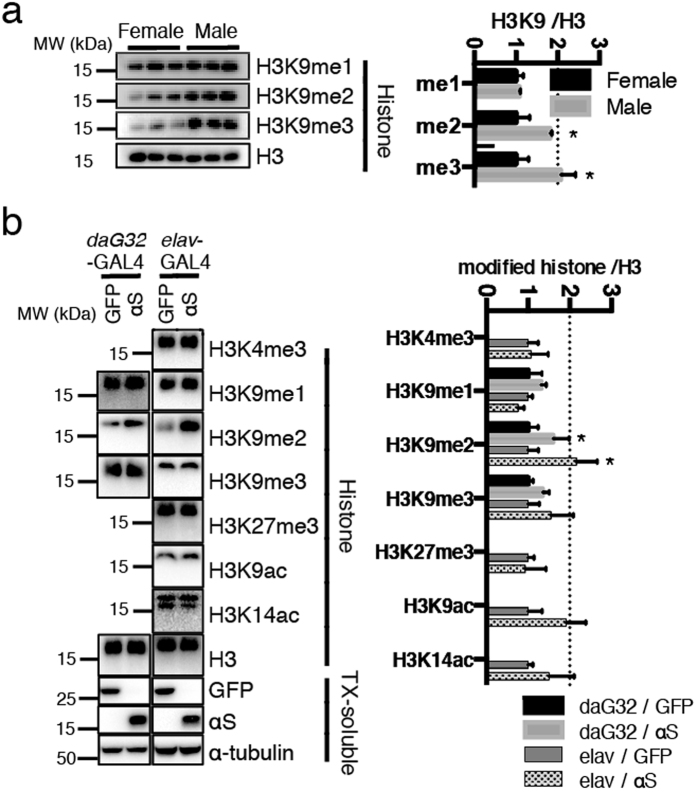
Analyses of modified histones in *Drosophila melanogaster*. Histones were extracted from 15 fly head tissues. Then, 3 different samples from each group were subjected to SDS-PAGE and Western blot analysis. (**a**) H3K9me2 and H3K9me3, representative marks of heterochromatin, were more prominent in males. *P < 0.05 by Sidak after Two-way ANOVA against female (n = 3). The result was shown as mean ± SEM. (**b**) Histones and Triton X-100 (TX) soluble fractions were analyzed by Western blotting. Green fluorescence protein (GFP) expressing flies are compared to αS transgenic *Drosophila*. Western blots from histone fractions of ubiquitous *daG32*-GAL4 driven transgenic male flies were probed for mono-methylated (H3K9me1), di-methylated (H3K9me2), tri-methylated (H3K9me3) as well as total histone H3. In addition to the aforementioned probings, the histone fractions from neuron-specific *elav*-GAL4 driven flies were probed for H3 tri-methylated at lysine-4 (H3K4me3) and lysine-27 (H3K27me3), as well as H3 acetylated at lysine-9 (H3K9ac) and lysine-14 (H3K14ac). Transgenic expressions of GFP and αS were confirmed in the whole lysates (TX-soluble), as well as α-tubulin as loading control. Band intensities were quantified by densitometric scanning; *P < 0.05 by Sidak after two-way ANOVA against GFP. The result was shown as mean ± SEM.

As the basic chromatin structure including most histone modifications are well conserved from human to flies, we used a panel of antibodies against modified histone H3 in Western blot analyses of chromatin extracted from head tissue of αS transgenic *Drosophila melanogaster*^21^. We first compared the H3K9 methylations among female and male flies. Due to sex chromosome dosage compensation, the genders differ in their epigenetic landscape. Specifically, in males the Y chromosome is extensively silenced in heterochromatin^22^. Indeed chromatin isolated from male fly heads showed more H3K9 di- and tri-methylations than that from females (Fig. 1a). This initial technical control experiment confirmed that the methodology is suitable for the proper detection of histone modifications in *Drosophila*.

First we examined transgenic flies expressing wt-αS ubiquitously under control of a *daG32*-GAL4 driver. Compared with GFP controls, H3K9 methylations were enhanced in male fly heads expressing αS (Fig. 1b). Densitometric quantification of the Western blot band strengths revealed significant differences for H3K9me2. Likewise, expressing αS with a pan-neuronal *elav*-GAL4 driver enhanced selectively the H3K9me2 signals in fly heads (Fig. 1b). Histone H3 methylations at other sites (H3K4me3, H3K27me3) showed no significant differences, and enhanced acetylations (H3K9ac, H3K14ac) did not reach statistical significance (Fig. 1b). Interestingly, the altered H3K9 methylations were detected in male but not in female flies (data not shown). Due to the enormous complexity of H3K9me biology^23^, it is hard to draw straightforward conclusions from this pilot experiment. Nevertheless, it is remarkable that αS is potentially capable of elevating the amount of H3K9me2 even at the level of whole tissue chromatin. To gain further insight into αS-mediated histone modifications, we turned to a cell culture model.

### Enhanced H3K9 methylations upon αS induction in retinoic acid differentiated neuroblastoma cells

**Figure 2:**
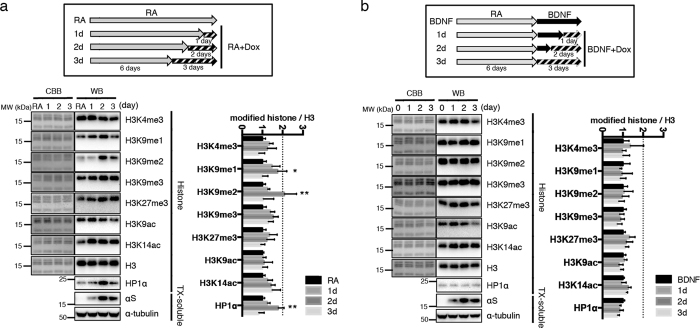
Histone modifications in SH-SY5Y cells. Western blots are presented in ‘WB’ columns, and Coomassie Brilliant Blue stained gels after the transfer are shown in ‘CBB’ columns. (**a**) Inducible αS expressing cells were treated with 10 nM RA in medium supplemented with 3% fetal bovine serum for 6 days followed by the addition of doxycycline (Dox). After further incubation for 1–3 days, histones and Triton X-100 (TX)-soluble fractions were analyzed by Western blotting. After two days induction of αS, levels of H3K9me1, H3K9me2 and HP1α were significantly elevated. *P < 0.01 by Sidak after Two-way ANOVA against 0 day; **P < 0.001 against 0, 1 and 3 day (n = 9). The result was shown as mean ± SEM. The experiments were repeated six times. (**b**) Cells were treated with 10 nM RA for 6 days, then further incubated with 50 ng/ml BDNF for 3 days with αS induction (0–3 days). Histones and TX-soluble fractions were subjected to Western blotting. No significant changes were observed by Sidak after Two-way ANOVA (n = 7). The result was shown as mean ± SEM. The experiments were repeated three times.

To study the effects of αS expression on histone modifications and epigenetic effects in cell culture, we used human neuroblastoma SH-SY5Y clones in which wt-αS can be induced by a tetracycline-controlled transcriptional activation system^24^. In order to recapitulate the non-dividing property of neuronal cells, we investigated epigenetic effects of αS induced in SH-SY5Y cells pre-differentiated by retinoic acid (RA) and fully differentiated by RA plus brain-derived neurotrophic factor (BDNF). In each experiment, cells were collected after αS induction by adding doxycycline to the culture media for 0 to 3 days. αS induction started within 1d doxycycline treatment and reached high expression levels after 2 and 3 days (Fig. 2; TX-soluble).

Cells cultured with 10 nM RA for 6 to 8 days, which leads to complete growth arrest^25^, were additionally treated with 1 μg/mL doxycycline for 1 to 3 days (Fig. 2a). The amounts of methylated H3K9 species in the chromatin extracts increased over a period of 1–2 days and returned to baseline at day 3 (Fig. 2a). Elevation of HP1α, which attaches and stabilises H3K9me2/3^26^, followed the same time course (Fig. 2a). Meanwhile, other methylation and acetylation marks did not change significantly. H3K9 methylation changes were exclusively detected in cells treated only with RA. In BDNF-differentiated cells the H3K9 methyl marks were not significantly altered by αS induction (Fig. 2b). Doxycycline itself did not affect the methylation status of H3K9 (Supplementary Fig. 1a). Induction of the PD mutant A53T-αS^2^ had similar effects as wt-αS (Supplementary Fig. 1b), suggesting that high αS protein levels are a more important driving force than mutated species for the observed H3K9 methylations, at least in this cell culture model system.

### Up-regulation of EHMT2 after αS expression

**Figure 3:**
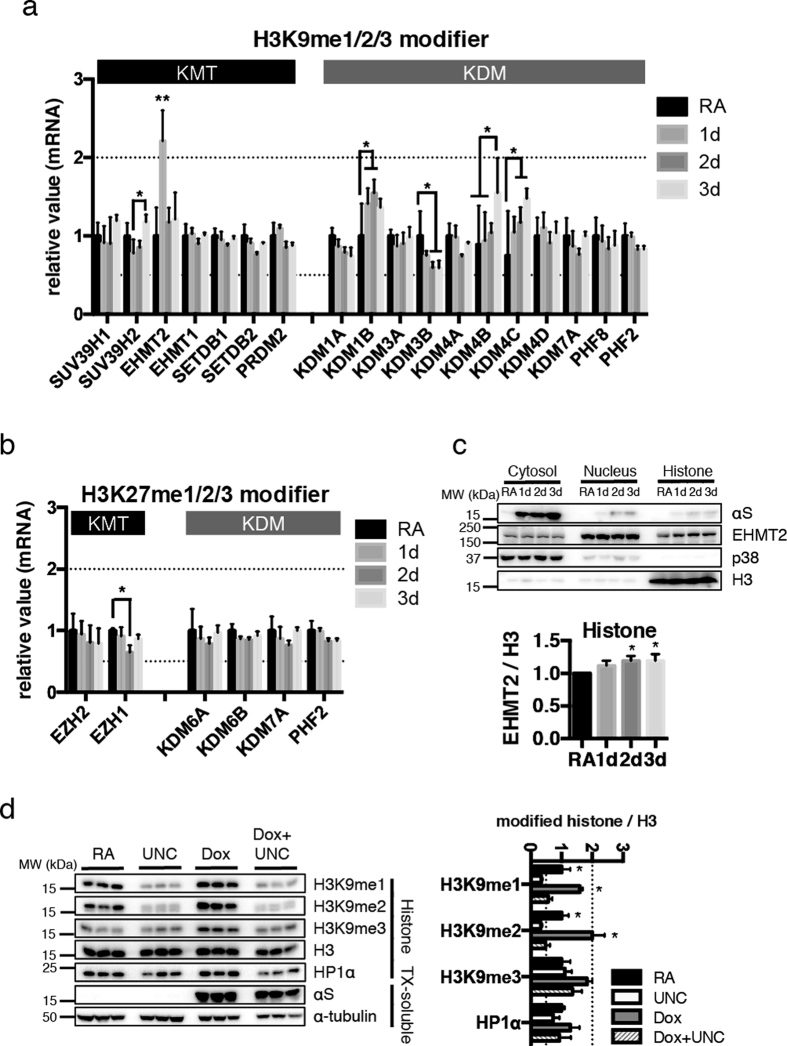
Analyses for histone modifying enzymes. (**a,b**) Cells were collected after RA pretreatment and doxycycline induction of αS for 1–3 days, as in Fig. 2a. Total mRNA was extracted, reverse transcribed and the obtained cDNA was analyzed by PCR using specific primer pairs (see Supplementary Table S1). (**a**) *EHMT2* was significantly upregulated at the first day of αS induction. Less prominently, *KDM1B*, *KDM4B*, and *KDM4C* were increased and *KDM3B* was decreased, but mostly at later time points trailing the observed H3K9 methylation changes. *P < 0.05; **P < 0.005 by Sidak after Two-way ANOVA (n = 4). The result was shown as mean ± SEM. (**b**) *EZH1* mRNA levels were mildly decreased after two days of αS induction. *P < 0.05 by Sidak after Two-way ANOVA (n = 4). The result was shown as mean ± SEM. (**c**) Cells were fractionated into cytosol, nucleus, and histone rich fraction, then EHMT2 protein levels were determined by Western blotting. p38 was used as a marker for cytosolic fraction, and H3 for histone. EHMT2 showed mild but significant increase in the histone fraction upon αS induction. *P < 0.01 against naive by Dunett after one-way ANOVA (n = 4). The result was shown as mean ± SEM. (**d**) After six days incubation with RA and αS induction by doxycycline, where indicated, some cells were further treated with 5 μM UNC0638, EHMT inhibitor. Histone and TX-soluble fractions were prepared and probed for the lysine-9 methylated forms and total H3 as well as HP1α. Induction of αS was confirmed in the TX-soluble fraction, as well as α-tubulin as loading control. *P < 0.005 against RA with UNC, or Dox with UNC by Sidak after Two-way ANOVA (n = 3 as shown). The result was shown as mean ± SEM. The experiments were repeated three times.

Histone methylation status is tightly regulated by lysine methyl transferase (KMT) and lysine demethylase (KDM) enzymes. To clarify the enzymatic regulation of the H3 methylation states through αS with RA, transcripts of candidate KMTs and KDMs were analyzed by RT-PCR. Among the H3K9 modifiers, most significant changes were detected in *EHMT2* transiently at 1 day after αS induction (Fig. 3a). Some KDMs showed a little alteration mainly at the later time points. On the H3K27 modifiers, *EZH1* transcript modestly declined over the αS induction time course (Fig. 3b), but the corresponding methyl mark H3K27me3 was not significantly altered at the whole chromatin level during the observation period (Fig. 2a).

Also at the protein level, EHMT2 gradually increased in the histone fraction (Fig. 3c). To further test the role of EHMT2 on the H3K9 methylation in our model, the chemical inhibitor for EHMT, UNC0638^27^ was tested. As expected, cells treated with a saturating dose of 5 μM UNC0638 showed reduced H3K9me1 and H3K9me2 levels (Fig. 3d). On the contrary, UNC0638 did not affect the level of H3K9me3. This result supported the specificity of this inhibitor to EHMT2, which does not affect H3K9me3. Importantly, the elevated levels of both H3K9me1 and H3K9me2 levels were abolished by UNC0638 in αS expressing cells (Fig. 3d).

### Identification of αS-sensitive H3K9me2 target genes

**Figure 4:**
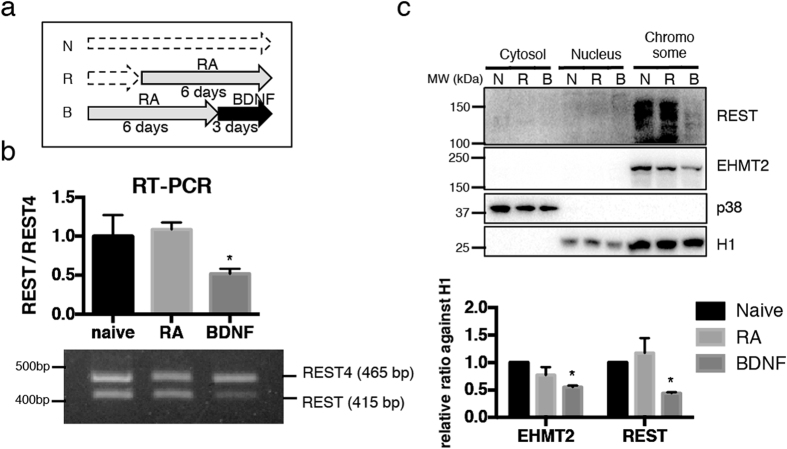
Alteration of REST expression levels during differentiation of SH-SY5Y cells. (**a**) SH-SY5Y cells were treated with 10 nM RA supplemented with 3% FCS for 0 (N: naive) to 6 days (R: RA) followed by 50 ng/mL of BDNF (B: BDNF). (**b**) *REST* transcripts were analyzed using primer pairs, which amplify both full length REST (415 bp) and non-functional splicing variant *REST4* (465 bp). *REST* levels against *REST4* were significantly decreased after BDNF treatment. *P < 0.01 against naive by Dunett after one-way ANOVA (n = 5). The result was shown as mean ± SEM. (**c**) Cells were divided into cytosol, nucleus, and chromosome-rich fraction. REST and EHMT2 protein levels were determined by Western blotting. p38 was used as a marker for cytosolic fraction, and H1 was nucleus and chromosome. *P < 0.01 against 0d by Sidak after Two-way ANOVA (*N* = 3). The result was shown as mean ± SEM. The experiments were repeated three times.

To identify specific genes regulated by H3K9me2 after αS induction, we focused on genes known to be regulated by REST, which interacts with EHMT2, and consequently organizes the di-methylation of H3K9^28^. During the process of neuronal differentiation, downregulation of REST is an essential factor for introducing cell type specific gene expression patterns^29^. In contrast to previous reports indicating that REST is reduced in embryonic stem cells incubated with RA^30^, we observed that RA treatment alone was not sufficient to alter REST expression in SH-SY5Y neuroblastoma cells, but required full differentiatiation with BDNF to reduce REST mRNA levels (Fig. 4a,b). Concurrent with the dramatic reduction of REST protein levels at the chromosomal fraction in BDNF-differentiated cells, EHMT2 level was also declined (Fig. 4c). Alternative splicing of REST generates full length REST and a truncated form (called REST4) that results in a protein with less DNA binding ability and loss of repressive function^31^. We assessed these REST forms using RT-PCR with primer pairs enable to distinguish these variants^32^, and found low REST/REST4 ratio after incubation with BDNF (Fig. 4b). BDNF associated REST reduction was probable, whereas it is also documented that REST level was sustained during RA treatment. If a REST-EHMT2 pathway potentially acting on H3K9 methylation might be robustly suppressed in fully differentiated SH-SY5Y cells, at least a part of globally increased H3K9me2 during RA treatment alone happens to be conducted by REST complex through EHMT2 stimulation.

**Figure 5:**
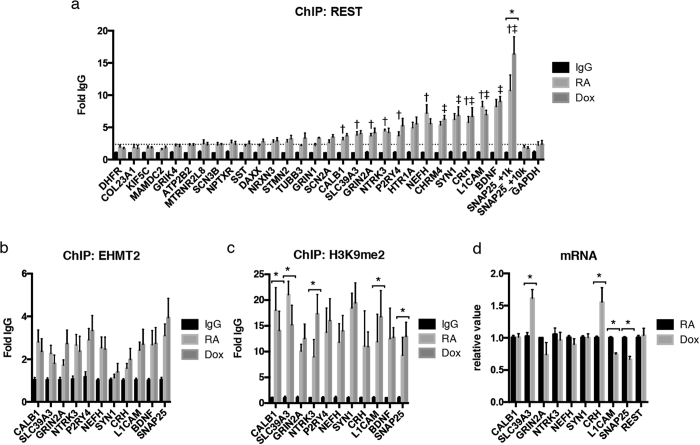
Identification of H3K9me2 target genes. (**a**) αS-inducible SH-SY5Y cells were treated with RA for 7 days, followed by αS induction with or without Doxycycline for two days (Dox or RA). Known REST-regulated genes were analyzed by chromatin immuoprecipitation (ChIP) using anti-REST antibodies. Eleven genes were significantly occupied with REST compared with GAPDH. ^†^*P* < 0.05, RA against RA of GAPDH, ^‡^*P* < 0.05, Dox against Dox of GAPDH by Dunn’s multiple comparison test after non-parametric one-way ANOVA (n = 4). After induction of αS, binding property of REST to RE1 of *SNAP25* (SNAP25_+1k) was significantly potentiated. *P < 0.05 by Sidak after two-way ANOVA (n = 4). IgG: IgG control; RA: cells treated with RA for 9 days. (**b**) ChIP analyses by EHMT2. Chromatin was obtained from same condition as (**a**). (**c**) ChIP analyses by H3K9me2. In the presence of αS, *NTRK3*, *L1CAM*, and *SNAP25* genes were highly occupied with H3K9me2. *P < 0.05 by Sidak after two-way ANOVA (n = 4). (**d**) RT-PCR analysis of mRNA expression under the same conditions as above was performed. Transcripts levels of *L1CAM* and *SNAP25* were significantly decreased after αS induction. *P < 0.05 by Sidak after two-way ANOVA (n = 5). The result was shown as mean ± SEM.

Genomic DNA fragments purified from chromatin immunoprecipitation (ChIP) using REST antibodies were analyzed by specific primer pairs amplifying 29 candidates. Eleven genes were significantly occupied with REST in a statistical comparison with *GAPDH* (Fig. 5a). The highest peak appeared on the RE1 binding site (SNAP25_+1k) of *SNAP25*, and it was distinct from distal site (SNAP25_+10k) (Fig. 5a). RE1 occupancy of *SNAP25* was further increased after αS induction. The strongest RA-responsive RE1 genes using the same primer pairs as for REST were selected for ChIP analyses with EHMT2 and H3K9me2 (Fig. 5b and c). After αS induction, H3K9me2 was significantly enriched at RE1 site of three genes, encoding the neurotrophin-3 receptor TrkC (*NTRK3*), the L1 neural cell adhesion molecule (*L1CAM*), and the synaptosomal-associated protein SNAP25 (Fig. 5c). EHMT2 ChIP showed similar trends (Fig. 5b). In addition, promoters of calbindin (*CALB1*) and a zinc transporter (*SLC39A3*) showed significantly reduced RE1 site promoter occupancy of H3K9me2 (and the same trend for EHMT2) after αS induction.

The H3K9me2 mark is indicative for facultative heterochromatin resulting in transcriptional repression. Accordingly, *L1CAM* and *SNAP25* mRNA levels were reduced after αS induction (Fig. 5d). Epigenetic silencing of the expression of the neurotrophin-3 receptor TrkC (*NTRK3*) was not confirmed by RT-PCR (Fig. 5d). Conversely, mRNA expression of the zinc transporter SLC39A3 (but not *CALB1*) was enhanced after αS induction (Fig. 5d), consistent with reduced promoter occupancy with the repressive H3K9me2 mark (Fig. 5c). Finally, mRNA expression of the corticotropin release hormone (*CRH*) was enhanced after αS induction (Fig. 5d), without significant changes in H3K9me2 promoter occupancy (Fig. 5c).

**Figure 6:**
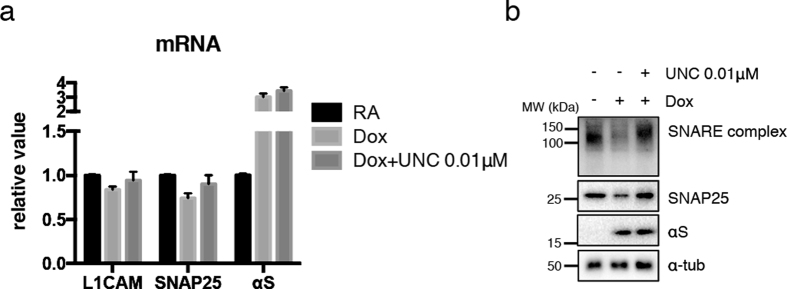
The effect of low dose EHMT inhibitior in αS-mediated mRNA and protein reduction. (**a**) αS-inducible SH-SY5Y cells were treated with RA for 7 days, then further cultured with or without Dox for 2 days (Dox or RA). Additional treatment with 0.01 μM of UNC0638 (Dox+UNC 0.01 μM) alleviated the suppressed mRNA expression of *L1CAM* and *SNAP25*. (**b**) Samples for Western blotting were collected in same condition as (**a**). Decreased protein levels of SNAP25 were recovered after adding EHMT inhibitor, UNC0638. Higher molecular SNAP25-immunopositive bands representing SNARE complex went along with SNAP25 monomer bands.

To confirm that the apparent epigenetic silencings of *L1CAM* and *SNAP25* expression in response to αS induction were indeed mediated by EHMT-catalysed H3K9 methylation, we tested the EHMT inhibitor UNC0638. At a relatively low dose, which does not occlude small effects due to strong loss of H3K9me2, UNC0638 could mitigate the altered transcript levels of *L1CAM* and *SNAP25* (Fig. 6a). Thus, αS regulated H3K9me2 epigenetic regulation of L1CAM and SNAP25 is sensitive to pharmacological inhibition of the H3K9 methyltransferases EHMT. Reduced protein levels of SNAP25 were also recovered after EHMT inhibition (Fig. 6b), also when examining SNAP25 associated with SNARE complex in the upper part of the gel. Thus, overexpression of αS enhances H3K9me2 at the *SNAP25* gene, which may repress its expression epigenetically and thus might affect SNARE complex assembly and hence, synaptic functions.

## Discussion

The previous study of Kontopoulos *et al*. used a nuclear-targeted αS fusion protein to document histone H3 acetylation dependent neurotoxicity^11^, and Outeiro *et al*. showed that the histone deacetylase sirtuin-2 modulates αS toxicity^12^. In the present investigation, we systematically screened a panel of modified histone antibodies for steady-state level changes in transgenic *Drosophila* and in αS inducible neuroblastoma cells and found that αS rather selectively enhances H3K9 mono- and di-methylation. The H3K9me1/2 methyltransferase EHMT2 is involved in this process. Such epigenetic silencing affects the neural cell adhesion molecule L1 and the synaptosomal-associated protein SNAP25. The resulting reduction of the SNARE complex component SNAP25 might act within a feedback network to tone synaptic vesicle release regulated by the lipid chaperone αS, and in pathological conditions this mechanism could derail and contribute to synaptic dysfunction occurring in PD.

Each histone modification has unique roles in the cell, and directly regulates the transactivation or repression of particular genes in appropriate periods for proper functioning of cells. With the advance of molecular genetics, several histone modifications have been associated with particular transcriptional states, and methylated H3K9 is a well-characterized mark as a representative signature for heterochromatin in *Drosophila* as well as in human. At the first step of this study, we could observe increased histone marks associated with heterochromatin in male flies. Using this evaluation system, we explored the epigenetic events through αS, and found elevated signals of di-methylated H3K9 in male transgenic flies. The fact that *elav*-GAL4 (neuron specific) driver showed similar effects on this specific histone modification as *daG32*-GAL4 (systemic) is somewhat premature to conclude neuron autonomous mechanisms, but at least it suggest that neurons would be more sensitive for epigenetic events exerted by αS. Indeed, similar epigenetic events were successfully reproduced in experimental neuronal cells.

Histone methylation states are regulated by sets of enzymes carrying opposing function: methyl-attaching enzyme (histone lysine methyl transferase) and -detaching enzyme (histone lysine demethylase). We systematically analyzed by RT-PCR the mRNA expressions of these enzymes and found some transcriptional changes in H3K9 methylation modifiers. Among them, *EHMT2* was the most prominent effector because it elevated at earlier phase and reached levels 2-fold over basal. EHMT2 protein levels also showed mild but significant increase in histone-rich fraction. Enzymatic activity of human EHMT2 is selective toward H3K9me1 and H3K9me2^18^, in line with di-methylated H3K9 being the most significant altered mark in response to αS. These results suggest that EHMT2 plays a central role in H3K9 methylation in our model. Indeed, the chemical inhibitor for EHMT, UNC0638^27^ could reverse the EHMT2 associated histone marks, and this result might support the reliability of this pathway.

Interestingly, overexpression of αS in BDNF treated cells failed to modulate the methylation status at H3K9, whereas in RA-treated cells a transient increase in H3K9me1 and H3K9me2 was observed after αS induction. It appears that BDNF has inhibitory effects for αS-mediated H3K9 methylations, perhaps at least in part involving reduced levels of the REST and EHMT2 in the chromatin fraction. REST is a transcription factor first described as a repressor for the neuronal genes in non-neuronal cells^33^, but later studies indicated that REST governs neurogenesis, or neural plasticity^30^, and more recently, decreased levels of REST in the tissues of frontal cortex from Alzheimer’s disease patients were documented^34^. These findings imply the role of REST in maintaining neuron and neuronal system. EHMT2 is also known to be involved in neuronal plasticity^16^, and its expression was affected by various conditions^18^. Notably, recent report documented that RA, or 12-*O*-tetradecanoylphorbol-13-acetate (TPA), which attributes to cell differentiation in leukemic cells, activates EHMT2 along with certain transcriptional factor resulting in the suppression of target genes^35^,^36^. Similar with these EHMT2 regulations, we also found that EHMT2 levels were increased after αS induction during RA treatment. Therefore, we speculate that αS induction during RA treatment arouses potential functional interaction between REST and EHMT2.

Although many REST target genes were postulated, only a small number of genes were validated in cultured human cells^19^,^37^. We assessed a number of REST target genes, and found that the RE1 site of *SNAP25* gene was significantly occupied with H3K9me2. SNAP25 forms a part of SNARE complex as t-SNARE, and is important to maintain synaptic functions^38^. Several studies have concerned about the role of αS in SNARE complex formation and functioning. Knockout mouse studies suggested that αS acts as a lipid chaperone to support the function of SNARE^39^,^40^, but studies in αS mutant mice showed SNARE complex disturbances^41^,^42^. It appears that αS effects on SNARE complex assembly and function are intricately controlled, and we suggest that αS-mediated epigenetic regulation involving H3K9me2 participates in this molecular network.

Here we show that alterations induced by αS overexpression lead to changes in the transcriptional regulation of genes. To illustrate this process, the paradigm that αS interacts directly with histone modifiers would be attractive, but we could not show the direct interaction between αS and EHMT2 in nuclear fraction (data not shown). However, cytosolic αS might be as important to affect histone marks. For instance, enzymatic activity of the histone acetyltransferase p300 was disturbed by cytosolic αS^43^. Similarly, in our experiment, the vast majority of αS was located in the cytosolic fraction. We cannot exclude the possibility that small amounts of nuclear αS have an effect on histone marks directly, but some intermediate molecules are most likely bridging αS and nuclear proteins. Linking αS overexpression to epigenetic alterations is still at is early steps, but our study provides a new hint to be followed up.

**Figure 7:**
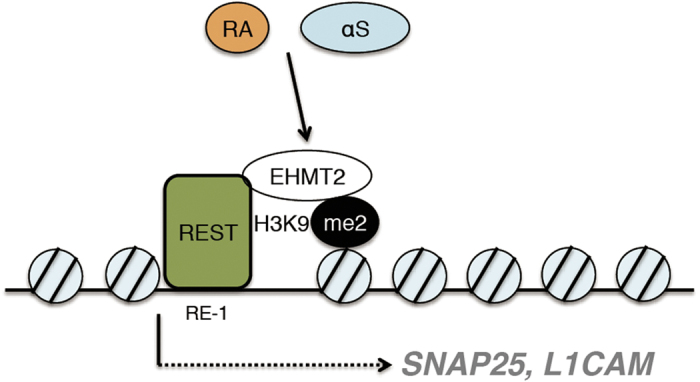
Graphical abstract. In the presence of RA, in part of EHMT2 level is positively regulated by αS. Cooperative with transcription factor REST, which directly binds to target gene promoter at RE-1, EHMT2 catalyzes di-methylation (me2) of H3K9 resulting in repression of *SNAP25* and *L1CAM* transcription.

In conclusion, we show enhanced H3K9 mono- and di-methylation through αS in *Drosophila* and SH-SY5Y cells. In this process, EHMT2 might be a key regulator for this modification. Here, we investigated REST target genes harboring RE1 sites, and found the promoter region of *SNAP25* occupied with H3K9me2 upon overexpression of αS resulting in reduced gene expression and ultimately lower protein levels (Fig. 7). As the experimental models we used were specified to investigate the physiological role of αS, it may not always connect with the neurodegenerative steps of human synucleinopathies. We revealed a new aspect of αS as its overexpression alters the distribution of histone marks on genes associated with the REST complex resulting in disturbed synaptic activities.

## Materials and Methods

### Fly stocks

All *Drosophila melanogaster* stocks were maintained on standard cornmeal-yeast agar-based fly food. Experiments were performed at 25 °C. The driver lines, *elav*-GAL4 and *daG32*-GAL4 were obtained from the Bloomington Drosophila Stock Center. Stable green fluorescent protein (GFP), or αS expression in neuron was achieved by recombination of the *elav*-GAL4 driver with UAS:GFP, or UAS:αS. For the whole body expression, the *daG32*-GAL4 driver was used for the recombination. Eight to 12 days old flies were sacrificed, then subjected to the histone modification analyses.

### Cell culture and differentiation

Wt-αS inducible SH-SY5Y cells were grown in Dulbecco’s modified Eagle’s medium (DMEM) supplemented with 10% FCS at 37 °C in 5% CO_2_. For the differentiation, 10 nM retinoic acids (SIGMA) were added to media supplemented with 3% FCS and cells were cultured for 6 days. In some experiments, full neuronal differentiation was achieved by treatment with 50 ng/ml BDNF (PeproTech). Expression of αS was induced by addition of 1 μg/ml doxycycline (Dox; SIGMA) to the media. EHMT inhibition was performed with UNC0638 (Tocris Bioscience).

### Histone extraction

Cells, or fly heads were washed twice with ice-cold PBS, then lysed in PBS containing 0.5% Triton X-100 (v/v), 0.02% NaN_3_ (w/v), and complete protease inhibitor (Roche) on ice for 10 minutes. Lysates were cleared by centrifuging at 390 × *g* for 10 minutes at 4 °C. Then, the supernatants were stored as Triton X soluble fraction (TX-soluble). The pellets were washed with same buffer, followed by 4 h incubation with 0.2N HCl. After centrifugation at 390 × *g* for 10 min, histone enriched fractions were obtained.

### Chromatin isolation

The procedure was performed as described previously^44^. Briefly, collected cells were resuspended in 10 mM HEPES, pH 7.9, 0.1% Triton X-100, 10 mM KCl, 1.5 mM MgCl_2_, 0.34 M sucrose, 10% glycerol, 1 mM dithiothreitol (DTT). After centrifugation at 1300 × *g*, supernatant was stored as crude cytosolic fraction, and then clarified by 20 000 × *g* centrifugation. Pellets were incubated with 3 mM EDTA, 0.2 mM EGTA, 1 mM DTT, then separated by 1700 × *g* centrifugation to obtain the nuclear fraction. Pellets were lysed in RIPA buffer, then the chromatin-rich fraction was isolated from the supernatant. In some experiments, 0.2N HCl was used instead of RIPA to obtain the histone fraction.

### Western blot analysis

Denatured protein was separated on polyacrylamide gels and transferred onto Hybond-P polyvinylidene difluoride membrane (Millipore). Membranes were incubated with 0.4% paraformaldehyde in PBS for 30 min, followed by blocking with 5% skim-milk in TBS-Tween 20, and then incubated with primary antibody in Western Blocking Reagent (Roche Applied Science) at 4 °C overnight. After incubation with HRP-conjugated secondary antibodies for 1 h at room temperature, proteins were detected by the Immobilon Western chemiluminescent HRP substrate (Millipore) using ChemiDocMP (Bio-Rad). Loading amounts of the samples to the SDS-PAGE gels, concentration of primary and secondary antibodies, and exposure time at the CCD system was optimized to get linearity of the signal intensities to the absolute protein amounts.

### Antibodies

The antibodies for Western blot analysis used were: mouse anti-α-tubulin (1:10,000; Sigma, clone B512), rat anti-α-synuclein (1:100; clone 15G7), mouse anti-α-synuclein (1:1000; BD, Synuclein-1), rabbit anti-GFP (1:1000; Santa Cruz), rabbit anti-H3 (1:16000; abcam), rabbit anti-H3K4me3 (1:16000; Abcam), rabbit anti-H3K9me1 (1:16000; Abcam), rabbit anti-H3K9me2 (1:4000; CST), rabbit anti-H3K9me3 (1:16000; Abcam), rabbit anti-H3K27me3 (1:8000; Millipore), rabbit anti-H3K9ac (1:8000; Millipore), rabbit anti-H3K14ac (1:8000; EpiGentek), rabbit anti-H4 (1:16000; Abcam), rabbit anti-H4K12Ac (1:16000; Abcam), rabbit anti-HP1α (1:1000; CST), rabbit anti-EHMT2 (1:2000; abcam), rabbit anti-p38 (1:1000; CST), goat anti-REST (1:250; Santa Cruz) and goat anti-SNAP25 (1:1000; Santa Cruz). The HRP-conjugated secondary antibodies were from Jackson ImmunoResearch Laboratories (1:10,000). The antibodies for chromatin immunoprecipitation (ChIP) used were: rabbit anti-EHMT2 (10 μg; Millipore), mouse anti-H3K9me2 (4 μg; Abcam), and rabbit anti-REST (10 μg; Millipore).

### Chromatin immunoprecipitation

Cells after the treatment, culture media was removed and washed once with warm PBS, then cross-linked by 1% formaldehyde in DMEM for 10 min at room temperature with mild shaking. Cross-linking was quenched by 0.65 M glycine for 5 min incubation. Cells were collected by scraping on ice, then incubated with cell lysis buffer for ChIP (5 mM PIPES, pH 8.0, 85 mM KCl, 0.5% NP-40) for 10 min. After removal of the supernatant, pellets were resuspended with nuclear extraction buffer for ChIP (50 mM Tris-HCl, pH 8.0, 10 mM EDTA, 1% SDS) supplemented with complete protease inhibitor cocktail. Sonication was performed as 15 sec pulses followed by 45 sec rest periods at 50% output for 10 times. After the centrifuge at highest speed, clarified supernatants were stored as chromatin samples. Dilution buffer (16.7 mM Tris-HCl, pH 8.0, 167 mM NaCl, 1.2 mM EDTA, 0.01% SDS, 1.1% Triton X-100) supplemented with complete protease inhibitor cocktail was added to the 25 μg of chromatin (9:1) followed by pre-cleared with 50 μl Protein A sepharose beads (Millipore) supplemented with 1 μL of sonicated herring DNA (10 mg/mL, Clontech). Samples were incubated with control rabbit IgG, REST, EHMT2, or H3K9me2 antibodies at 4 °C overnight followed by 2 h incubation with 50 μl beads per sample. The protein A beads were collected with centrifugation at 850 × *g* for 2 min and washed four times with high salt wash buffer (50 mM HEPES, pH7.9, 500 mM NaCl, 1 mM EDTA, 0.1% SDS, 1% Triton X-100, 0.1% deoxycholate), and two times with TE (10 mM Tris-HCl, pH8.0, 1 mM EDTA). The protein A beads were incubated with 20 μg/μl of proteinase K for 2 h at 55 °C. Beads were further incubated overnight at 65 °C. Beads were removed by centrifugation of 10 000 × *g* for 1 min, then DNA fragments were purified by using QIAquick PCR Purification Kit (Qiagen). REST target genes proven by wet experiments from previous literatures^19^,^34^,^37^, and the genes occupied by both EHMT2 and REST from ChIP-Atlas database (http://chip-atlas.org) based on registered ChIP-seq were analyzed by specific primer pairs (Supplementary Table S2) using LightCycler 480. The individual threshold cycle (*Ct)*, which obtained from 4 different samples each condition, of target genes were standardized by *Ct* of corresponding input control. The fold-change in binding genes, relative to the IgG control, was standardized as same as describing at the RT-qPCR section.

### RNA extraction and RT-PCR

RNA was isolated from SH-SY5Y cell lines with the RNeasy Mini kit (Qiagen) according to the manufacturer’s instructions. 2000 ng of total RNA was reverse transcribed with oligo hexamer and random primer (1:1) using the Transcriptor High Fidelity cDNA Synthesis kit (Roche Applied Science). 1 μl of cDNA was used as template for transcription amplification in a 25 μl reaction with 0.1 μl of GoTaq polymerase (Promega), and 0.5 μM primers. Amplified PCR products were subjected to electrophoresis using a 2% agarose gel stained with Midori-Green (Nippon Genetics, Tokyo, Japan). Densitometry was performed on Image Lab software v5.0 (Bio-Rad). For the qPCR tests, SYBR-Green dye was used instead of Taq polymerase, then analyzed by LightCycler 480 (Roche). The individual *Ct*, which obtained from 5 different samples each condition, of target genes were standardized by *Ct* of corresponding GAPDH. The fold-change in transcriptional level, relative to the RA treated control, was standardized by *ΔΔCt* method^45^. Primer pairs used are listed in Supplementary Table S1 and S3.

### SNARE complex analysis

Detection of SNARE complex was performed as described elsewhere^46^. Briefly, SH-SY5Y cells were scraped in buffer containing 10 mM HEPES pH 7.4, 200 mM sucrose 10 mM EDTA and 2 mM EGTA, then centrifuged at 600 × *g* for 5 minutes. The supernatant was mixed with one-fourth volume of 5x Laemmli buffer containing 315 mM Tris-HCl pH 6.8, 10% SDS, 50% glycerol, 0.05% BPB, and 100 mM DTT, followed by pulsatile sonication for 15 seconds to disperse the pellet. Samples were separated by 7.5% SDS-PAGE gel, then analyzed by Western blotting with anti-SNAP-25 antibodies.

## Additional Information

**How to cite this article**: Sugeno, N. *et al*. α-Synuclein enhances histone H3 lysine-9 dimethylation and H3K9me2-dependent transcriptional responses. *Sci. Rep*. **6**, 36328; doi: 10.1038/srep36328 (2016).

**Publisher’s note:** Springer Nature remains neutral with regard to jurisdictional claims in published maps and institutional affiliations.

## Supplementary Material

Supplementary Information

## References

[b1] Spillantini M. G. . α-Synuclein in Lewy bodies. Nature 388, 839–840, doi: 10.1038/42166 (1997). 9278044

[b2] Polymeropoulos M. H. . Mutation in the alpha-synuclein gene identified in families with Parkinson’s disease. Science 276, 2045–2047 (1997). 9197268 10.1126/science.276.5321.2045

[b3] Eriksen J. L., Dawson T. M., Dickson D. W. & Petrucelli L. Caught in the act: alpha-synuclein is the culprit in Parkinson’s disease. Neuron 40, 453–456 (2003). 14642269 10.1016/s0896-6273(03)00684-6

[b4] Satake W. . Genome-wide association study identifies common variants at four loci as genetic risk factors for Parkinson’s disease. Nat Genet 41, 1303–1307, doi: 10.1038/ng.485 (2009). 19915576

[b5] Simon-Sanchez J. . Genome-wide association study reveals genetic risk underlying Parkinson’s disease. Nat Genet 41, 1308–1312, doi: 10.1038/ng.487 (2009). 19915575 PMC2787725

[b6] Xu W., Tan L. & Yu J. T. Link between the SNCA gene and parkinsonism. Neurobiol Aging 36, 1505–1518, doi: 10.1016/j.neurobiolaging.2014.10.042 (2015). 25554495

[b7] Maroteaux L., Campanelli J. T. & Scheller R. H. Synuclein: a neuron-specific protein localized to the nucleus and presynaptic nerve terminal. J Neurosci 8, 2804–2815 (1988). 3411354 10.1523/JNEUROSCI.08-08-02804.1988 PMC6569395

[b8] Burre J. The Synaptic Function of alpha-Synuclein. J Parkinsons Dis 5, 699–713, doi: 10.3233/JPD-150642 (2015). 26407041 PMC4927875

[b9] Bannister A. J. & Kouzarides T. Regulation of chromatin by histone modifications. Cell Res 21, 381–395, doi: 10.1038/cr.2011.22 (2011). 21321607 PMC3193420

[b10] Goers J. . Nuclear localization of α-synuclein and its interaction with histones. Biochemistry 42, 8465–8471, doi: 10.1021/bi0341152 (2003). 12859192

[b11] Kontopoulos E., Parvin J. D. & Feany M. B. Alpha-synuclein acts in the nucleus to inhibit histone acetylation and promote neurotoxicity. Hum Mol Genet 15, 3012–3023, doi: 10.1093/hmg/ddl243 (2006). 16959795

[b12] Outeiro T. F. . Sirtuin 2 inhibitors rescue alpha-synuclein-mediated toxicity in models of Parkinson’s disease. Science 317, 516–519, doi: 10.1126/science.1143780 (2007). 17588900

[b13] Litt M. D., Simpson M., Gaszner M., Allis C. D. & Felsenfeld G. Correlation between histone lysine methylation and developmental changes at the chicken beta-globin locus. Science 293, 2453–2455, doi: 10.1126/science.1064413 (2001). 11498546

[b14] Trojer P. & Reinberg D. Facultative heterochromatin: is there a distinctive molecular signature? Mol Cell 28, 1–13, doi: 10.1016/j.molcel.2007.09.011 (2007). 17936700

[b15] Gupta-Agarwal S. . G9a/GLP histone lysine dimethyltransferase complex activity in the hippocampus and the entorhinal cortex is required for gene activation and silencing during memory consolidation. J Neurosci 32, 5440–5453, doi: 10.1523/JNEUROSCI.0147–12.2012 (2012). 22514307 PMC3332335

[b16] Maze I. . Essential role of the histone methyltransferase G9a in cocaine-induced plasticity. Science 327, 213–216, doi: 10.1126/science.1179438 (2010). 20056891 PMC2820240

[b17] Shinkai Y. & Tachibana M. H3K9 methyltransferase G9a and the related molecule GLP. Genes Dev 25, 781–788, doi: 10.1101/gad.2027411 (2011). 21498567 PMC3078703

[b18] Shankar S. R. . G9a, a multipotent regulator of gene expression. Epigenetics 8, 16–22, doi: 10.4161/epi.23331 (2013). 23257913 PMC3549875

[b19] Ding N. . Mediator links epigenetic silencing of neuronal gene expression with x-linked mental retardation. Mol Cell 31, 347–359, doi: 10.1016/j.molcel.2008.05.023 (2008). 18691967 PMC2583939

[b20] Roopra A., Qazi R., Schoenike B., Daley T. J. & Morrison J. F. Localized domains of G9a-mediated histone methylation are required for silencing of neuronal genes. Mol Cell 14, 727–738, doi: 10.1016/j.molcel.2004.05.026 (2004). 15200951

[b21] Feany M. B. & Bender W. W. A Drosophila model of Parkinson’s disease. Nature 404, 394–398, doi: 10.1038/35006074 (2000). 10746727

[b22] Hoskins R. A. . Heterochromatic sequences in a Drosophila whole-genome shotgun assembly. Genome Biol 3, RESEARCH0085 (2002). 10.1186/gb-2002-3-12-research0085 PMC151187 12537574

[b23] Riddle N. C. . Plasticity in patterns of histone modifications and chromosomal proteins in Drosophila heterochromatin. Genome Res 21, 147–163, doi: 10.1101/gr.110098.110 (2011). 21177972 PMC3032919

[b24] Hasegawa T. . The AAA-ATPase VPS4 regulates extracellular secretion and lysosomal targeting of alpha-synuclein. PLoS One 6, e29460, doi: 10.1371/journal.pone.0029460 (2011). 22216284 PMC3245276

[b25] Encinas M. . Sequential treatment of SH-SY5Y cells with retinoic acid and brain-derived neurotrophic factor gives rise to fully differentiated, neurotrophic factor-dependent, human neuron-like cells. J Neurochem 75, 991–1003 (2000). 10936180 10.1046/j.1471-4159.2000.0750991.x

[b26] Eissenberg J. C. & Elgin S. C. HP1a: a structural chromosomal protein regulating transcription. Trends Genet 30, 103–110, doi: 10.1016/j.tig.2014.01.002 (2014). 24555990 PMC3991861

[b27] Vedadi M. . A chemical probe selectively inhibits G9a and GLP methyltransferase activity in cells. Nature chemical biology 7, 566–574, doi: 10.1038/nchembio.599 (2011). 21743462 PMC3184254

[b28] Ooi L. & Wood I. C. Chromatin crosstalk in development and disease: lessons from REST. Nat Rev Genet 8, 544–554, doi: 10.1038/nrg2100 (2007). 17572692

[b29] Lunyak V. V. & Rosenfeld M. G. No rest for REST: REST/NRSF regulation of neurogenesis. Cell 121, 499–501, doi: 10.1016/j.cell.2005.05.003 (2005). 15907461

[b30] Ballas N., Grunseich C., Lu D. D., Speh J. C. & Mandel G. REST and its corepressors mediate plasticity of neuronal gene chromatin throughout neurogenesis. Cell 121, 645–657, doi: 10.1016/j.cell.2005.03.013 (2005). 15907476

[b31] Palm K., Metsis M. & Timmusk T. Neuron-specific splicing of zinc finger transcription factor REST/NRSF/XBR is frequent in neuroblastomas and conserved in human, mouse and rat. Brain Res Mol Brain Res 72, 30–39 (1999). 10521596 10.1016/s0169-328x(99)00196-5

[b32] Wagoner M. P. . The transcription factor REST is lost in aggressive breast cancer. PLoS Genet 6, e1000979, doi: 10.1371/journal.pgen.1000979 (2010). 20548947 PMC2883591

[b33] Schoenherr C. J. & Anderson D. J. The neuron-restrictive silencer factor (NRSF): a coordinate repressor of multiple neuron-specific genes. Science 267, 1360–1363 (1995). 7871435 10.1126/science.7871435

[b34] Lu T. . REST and stress resistance in ageing and Alzheimer’s disease. Nature 507, 448–454, doi: 10.1038/nature13163 (2014). 24670762 PMC4110979

[b35] Kim K. B. . H3K9 methyltransferase G9a negatively regulates UHRF1 transcription during leukemia cell differentiation. Nucleic Acids Res 43, 3509–3523, doi: 10.1093/nar/gkv183 (2015). 25765655 PMC4402520

[b36] Son H. J., Kim J. Y., Hahn Y. & Seo S. B. Negative regulation of JAK2 by H3K9 methyltransferase G9a in leukemia. Mol Cell Biol 32, 3681–3694, doi: 10.1128/MCB.00673–12 (2012). 22801367 PMC3430193

[b37] Bruce A. W. . Genome-wide analysis of repressor element 1 silencing transcription factor/neuron-restrictive silencing factor (REST/NRSF) target genes. Proc Natl Acad Sci USA 101, 10458–10463, doi: 10.1073/pnas.0401827101 (2004). 15240883 PMC478591

[b38] Chapman E. R., An S., Barton N. & Jahn R. SNAP-25, a t-SNARE which binds to both syntaxin and synaptobrevin via domains that may form coiled coils. J Biol Chem 269, 27427–27432 (1994). 7961655

[b39] Burre J. . Alpha-synuclein promotes SNARE-complex assembly *in vivo* and *in vitro*. Science 329, 1663–1667, doi: 10.1126/science.1195227 (2010). 20798282 PMC3235365

[b40] Chandra S., Gallardo G., Fernandez-Chacon R., Schluter O. M. & Sudhof T. C. Alpha-synuclein cooperates with CSPalpha in preventing neurodegeneration. Cell 123, 383–396, doi: 10.1016/j.cell.2005.09.028 (2005). 16269331

[b41] Garcia-Reitbock P. . SNARE protein redistribution and synaptic failure in a transgenic mouse model of Parkinson’s disease. Brain 133, 2032–2044, doi: 10.1093/brain/awq132 (2010). 20534649 PMC2892942

[b42] Nakata Y. . Accumulation of alpha-synuclein triggered by presynaptic dysfunction. J Neurosci 32, 17186–17196, doi: 10.1523/JNEUROSCI.2220-12.2012 (2012). 23197711 PMC6621870

[b43] Jin H. . alpha-Synuclein negatively regulates protein kinase Cdelta expression to suppress apoptosis in dopaminergic neurons by reducing p300 histone acetyltransferase activity. J Neurosci 31, 2035–2051, doi: 10.1523/JNEUROSCI.5634-10.2011 (2011). 21307242 PMC3041642

[b44] Wysocka J., Reilly P. T. & Herr W. Loss of HCF-1-chromatin association precedes temperature-induced growth arrest of tsBN67 cells. Mol Cell Biol 21, 3820–3829, doi: 10.1128/MCB.21.11.3820-3829.2001 (2001). 11340173 PMC87041

[b45] Livak K. J. & Schmittgen T. D. Analysis of relative gene expression data using real-time quantitative PCR and the 2(-Delta Delta C(T)) Method. Methods 25, 402–408, doi: 10.1006/meth.2001.1262 (2001). 11846609

[b46] Koticha D. K., McCarthy E. E. & Baldini G. Plasma membrane targeting of SNAP-25 increases its local concentration and is necessary for SNARE complex formation and regulated exocytosis. J Cell Sci 115, 3341–3351 (2002). 12140265 10.1242/jcs.115.16.3341

