# Supplementary material for: α-Synuclein enhances histone H3 lysine-9 dimethylation and H3K9me2-dependent transcriptional responses
Source: Sci Rep. 2016 Nov 3;6:36328. doi: 10.1038/srep36328 (PMC5093762; doi:10.1038/srep36328)
Supplement: Supplementary Information [file srep36328-s1.pdf]

SUPPLEMENTARY INFORMATION

α-Synuclein enhances histone H3 lysine-9 dimethylation and H3K9me2-dependent transcriptional responses

Naoto Sugeno, Sandra Jäckel, Aaron Voigt, Zinah Wassouf, Julia Schulze-Hentrich and Philipp Kahle

Supplementary figure legend

Figure S1. Doxycycline treatment to the parental cells, and inducible mutant αS cells. Western blots were presented in 'WB' column, and Coomassie Brilliant Blue stained gels after the transfer were shown in 'CBB' column. (a) Parental SH-SY5Y cells were treated with 10 nM RA in medium supplemented with 3% fetal bovine serum for 7 days followed by the addition of doxycycline (Dox). After further incubation for 2 days, histones and TX-soluble fractions were analyzed by Western blotting. No significant changes were observed by Two-way ANOVA (n=3). (b) Inducible A53T mutant αS expressing cells were treated with 10 nM RA in medium supplemented with 3% fetal bovine serum for 6 days followed by the addition of doxycycline (Dox). After further incubation for 1-3 days, histones and TX-soluble fractions were subjected to Western blotting. After two days induction of αS, H3K9me2 level was significantly elevated. *$P<0.01$ by Sidak after Two-way ANOVA against 0 day; (n=3). The experiments were repeated three times.

Figure S1

a

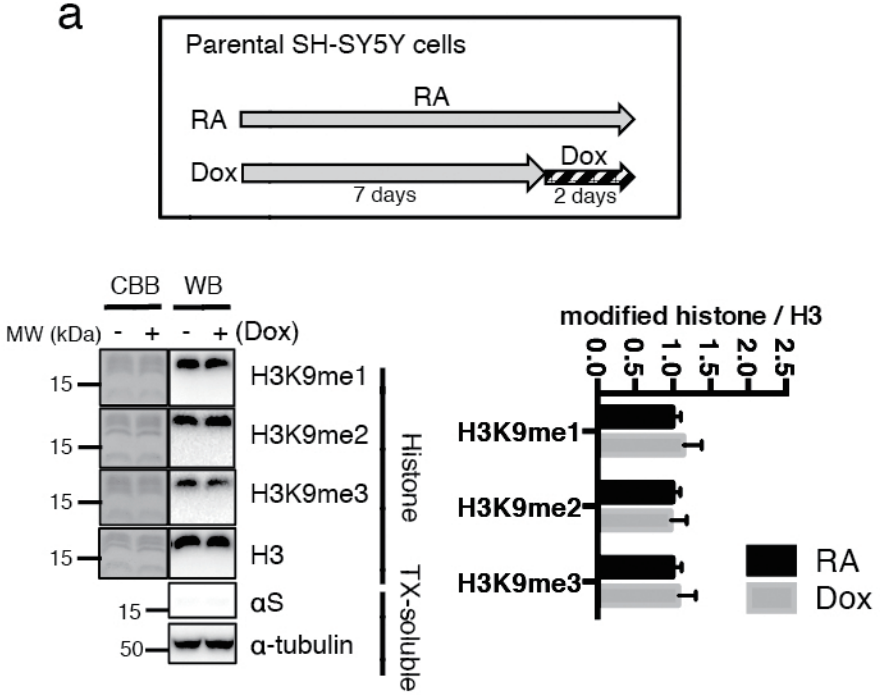
Parental SH-SY5Y cells
RA
RA
Dox
Dox
7 days
2 days
CBB
WB
MW (kDa)
- + - + (Dox)
15
15
15
15
15
50
H3K9me1
H3K9me2
H3K9me3
H3
αS
α-tubulin
Histone
TX-soluble
modified histone / H3
0.0
0.5
1.0
1.5
2.0
2.5
H3K9me1
H3K9me2
H3K9me3
RA
Dox

b

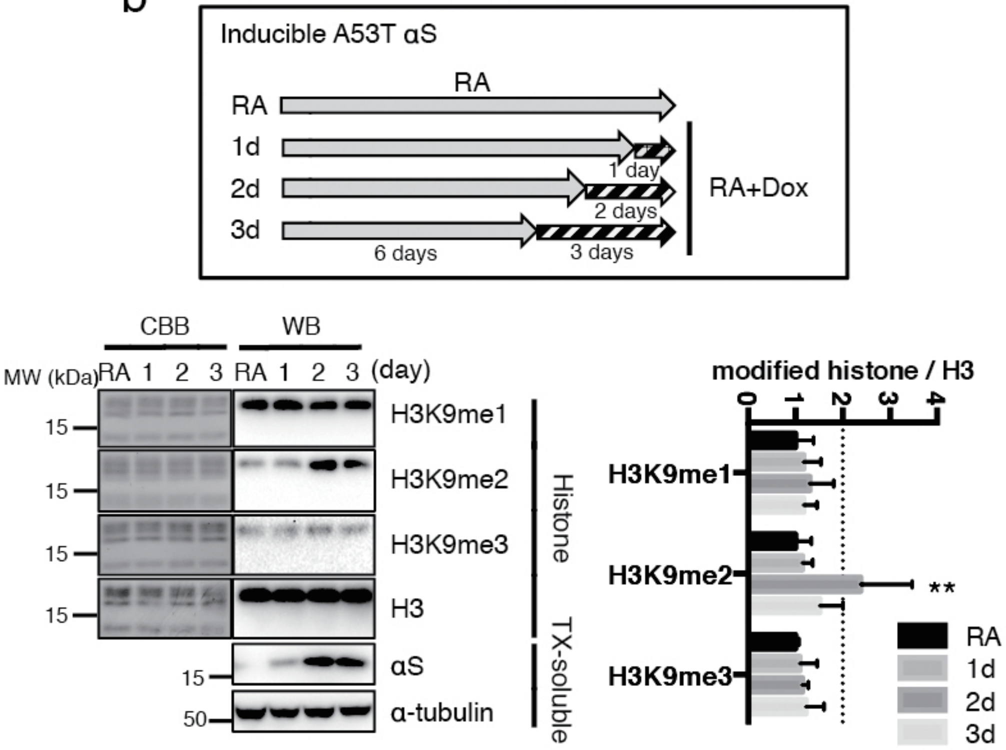
Inducible A53T αS
RA
RA
1d
2d
3d
1 day
2 days
6 days
3 days
RA+Dox
CBB
WB
MW (kDa)
RA 1 2 3 RA 1 2 3 (day)
15
15
15
15
15
50
H3K9me1
H3K9me2
H3K9me3
H3
αS
α-tubulin
Histone
TX-soluble
modified histone / H3
0
1
2
3
4
H3K9me1
H3K9me2
H3K9me3
**
RA
1d
2d
3d

Table. S1

RT-PCR primers

		**Forward**			**Reverse**	
KDM1A	5'-	atctgcagtccaaaggatgg	-3'	5'-	gccaacaatcacatcgtcac	-3'
KDM1B	5'-	tacggcaacatgtcctgtgt	-3'	5'-	tgggactaggttcggttttg	-3'
KDM3A	5'-	gccaacattggagaccactt	-3'	5'-	caccctgttggcaattcttt	-3'
KDM3B	5'-	caggaatttggagaccagga	-3'	5'-	gccagtccttgagtttgagc	-3'
KDM4A	5'-	ttgcttggcacactgaagac	-3'	5'-	tcagcattaacggggaaatc	-3'
KDM4B	5'-	gcctcaggaaccatcactgt	-3'	5'-	ccctgcgactctatgtaggc	-3'
KDM4C	5'-	gcttgcgagaaggtcatttc	-3'	5'-	gacttctccctcagcaggtg	-3'
KDM4D	5'-	aaatatgtacggggcaacca	-3'	5'-	tactcagacctgggggtacg	-3'
KDM6A	5'-	ctactggaagaatgctgcct	-3'	5'-	ctgaaaatgctttaaactag	-3'
KDM6B	5'-	cagcatctatctggagagca	-3'	5'-	gcctgtcagatcccagttct	-3'
KDM7A	5'-	ccccataccattcccgaaga	-3'	5'-	actgtggaggatggttggag	-3'
PHF8	5'-	tggatgaacaggacagcttg	-3'	5'-	gcagccaaacctgtctcaat	-3'
PHF2	5'-	tacgacgttacccgcttcat	-3'	5'-	ccaggtccgcttcttcttta	-3'
SUV39H1	5'-	gctatgactgcccaaatcgt	-3'	5'-	acacgtcctccacgtagtcc	-3'
SUV39H2	5'-	ggtgtgtgccttgcctagtt	-3'	5'-	aacgggcacttcagattttg	-3'
EHMT2	5'-	aggggtgtccaatgacacat	-3'	5'-	tctcacagagcaccatcagg	-3'
EHMT1	5'-	gggaaggaaaccttggagag	-3'	5'-	ggagagcgcttattctggtg	-3'
SETDB1	5'-	agacatctcctgccgtgact	-3'	5'-	gccgtgtagagcctcgatag	-3'
SETDB2	5'-	gagtggaatcagtgcccatt	-3'	5'-	ttgacaaggggaagttttg	-3'
EZH2	5'-	gaagtaaagagtatgtttag	-3'	5'-	catcttccaccataaaattc	-3'
EZH1	5'-	tccttcacccttttcatgcc	-3'	5'-	actatgtggtgccttctccg	-3'
PRDM2	5'-	gcttttcccttctgctgttg	-3'	5'-	aatgcacatccatcccaaat	-3'
REST&REST4	5'-	gagcgagtatcactggaggaaacattt	-3'	5'-	atagtcacatacagggcaattgaactgc	-3'
β-actin	5'-	ggacttcgagcaagagatgg	-3'	5'-	agcactgtgttggcgtacag	-3'

Table. S2

ChIP-qPCR primers

		Forward			Reverse	
BDNF	5'-	GTAAAGCCAACCCTGTGTCG	-3'	5'-	TCCGCTCCAAAATCTGACTC	-3'
CHRM4	5'-	AGACAAGCGCTGGAGCGGAATCA	-3'	5'-	AATCTCAGAGGTCGCTGGGAAC	-3'
GRIK4	5'-	AGTCAGTATGGACATAATAGCAGGT	-3'	5'-	TTTAAAGCAGGGGAACATCTCT	-3'
GRIN1	5'-	TGCGACCCCAAGATCGTCAA	-3'	5'-	CTTGTTGGCCTGGTTCACGG	-3'
GRIN2A	5'-	GTCTCCCCACTTGGAATTGA	-3'	5'-	AGTCCTTTGATCCGTGCTGT	-3'
L1CAM	5'-	GATAGCCAGGACGTTGGGTA	-3'	5'-	TGTCCCTGGTGCTGAAATCG	-3'
NEFH	5'-	CTCCCTTCCTTCCCCTAAAG	-3'	5'-	TGGCTCATGTTTTATGCTGTG	-3'
NTRK3	5'-	GCTCACCTTCCCCTAGCATTG	-3'	5'-	CAAGGCAGTGGCATTCGCT	-3'
NPTXR	5'-	GCCCAGAGGTAGAGGGGATA	-3'	5'-	GTGCCCACCACTTGTTTTG	-3'
NRXN3	5'-	TTATTTCCCCTTTCCCTTGG	-3'	5'-	AACCCTTTTCCATCCTCCAG	-3'
P2RY4	5'-	CTCCAAAGCCACCCACTACT	-3'	5'-	TCATCCACATCCCACTTGAA	-3'
SCN2A	5'-	AGCACCATGGACAGCGTTAC	-3'	5'-	CAGTTGTGAAGGCCAGGATCA	-3'
SNAP25	5'-	GGGTGCTATTATCCAGGGAAG	-3'	5'-	CAGGCGGCATAAATCAAGTC	-3'
SNAP25	5'-	AGTGGTTGTCGTGAGACCTA	-3'	5'-	CCCTGGAAATCTGGTATGGC	-3'
SYN1	5'-	GGTGCTGAAGCTGGCAGT	-3'	5'-	TGGGTTTTAGGACCAGGATG	-3'
STMN2	5'-	AAGGAGAGTGCCCTGCTATT	-3'	5'-	TAGCAGCTTTCTGTCCAATCACA	-3'
TUBB3	5'-	GGAGCCTTTGTCCTCCGC	-3'	5'-	CGCAGGTCTTTCAGGCGAG	-3'
KIF5C	5'-	AAGCCCCTTGACAGATCCAGT	-3'	5'-	TTGGTTGACGTGCAGCAAAT	-3'
MAMDC2	5'-	AATGGCATTGCTCCCGTGT	-3'	5'-	ACGGAGACAGAGAGTAGGGTG	-3'
COL23A1	5'-	CCATAGAGTGTTACCTGAGCGT	-3'	5'-	AACAGAAACATTCCATGGCCC	-3'
DHFR	5'-	CCTAGGGTTCGTCAAGTTCGTG	-3'	5'-	AAAACACTCTTTACGCCGGT	-3'
MTRNR2L8	5'-	TACTACCGAACAGCTTTAACCAAAT	-3'	5'-	CTTGCTGTATTATGCTTGGTTGC	-3'
CRH	5'-	ATGAGCTTAGGTGCGGAGC	-3'	5'-	CTACGGGACTGCCTTAGACG	-3'
CALB1	5'-	CTTTTGCTCACTCCCCCTCG	-3'	5'-	AATCACCGTCAGCGTCGAAA	-3'
SST	5'-	GATAAGCGCAGTCGGTCACA	-3'	5'-	GCTTCGTGCCCAGACAACTA	-3'
ATP2B2	5'-	ACATCCTGCTCTGTGTCAGC	-3'	5'-	TCAAGGGCACAGTGCTGATT	-3'
DAXX	5'-	CTTTTATGGCGCGTTGTGCT	-3'	5'-	TTTCCACGGCTTATTCGGCT	-3'
SCN3B	5'-	GAGAAGCAGTTAGGGCGGAC	-3'	5'-	GGTTGATTCACCTCTCGCCT	-3'
HTR1A	5'-	AGGTGGCGACATAAAACCTCA	-3'	5'-	CTTGGTGGAATCCCGGTTCG	-3'
SLC39A3	5'-	ATCTGCTTCTTCAGCCGTCG	-3'	5'-	AAGGGATTTCCAACGGAGGC	-3'
GAPDH	5'-	GAAAGGCAATCCCAGAAAGG	-3'	5'-	TCTAGCTAAAAGCCGGTTGC	-3'

Table. S3

RT-qPCR primers

		Forward			Reverse	
CALB1	5'-	TTTCGACGCTGACGGAAGT	-3'	5'-	TCTTTGCCCATACTGATCCACA	-3'
SLC39A3	5'-	AGGGAAAAGCTCCAGAAGGTC	-3'	5'-	CTCCAGGTCGATGAAGGACG	-3'
GRIN2A	5'-	GCACACCTTGCACCCATTTAT	-3'	5'-	TCAGGCTCAGCGTATGGTTC	-3'
NTRK3	5'-	ATGGAGCTCTACACCGGACT	-3'	5'-	GGTGAGCCGGTTACTTGACA	-3'
NEFH	5'-	CTGAGGAACACCAAGTGGGAG	-3'	5'-	GGAATTGGGCCAAAGCCAATC	-3'
SYN1	5'-	TTTGCCCAGATGGTTCGACT	-3'	5'-	CAACCTTGACCTTGCCCATC	-3'
CRH	5'-	TGGGAAGCGAGTGCCCCTA	-3'	5'-	GAAATCCAAGGGCTGAGGGTG	-3'
L1CAM	5'-	GCGGCAAATACTCAGTGAAGG	-3'	5'-	GGACCTGTACTCGCCGAAG	-3'
SNAP25	5'-	CAGTTGGCTGATGAGTCGCT	-3'	5'-	TTCATGCCTTCTTCGACACGA	-3'
REST	5'-	GAACTCATACAGGAGAACGCC	-3'	5'-	TGTCTTGCATGGCGGGTTAC	-3'
SNCA	5'-	AAATGTTGGAGGAGCAGTGG	-3'	5'-	TCCAGAATTCCTTCCTGTGG	-3'
GAPDH	5'-	CGGTTTCTATAAATTGAGCCCGC	-3'	5'-	TGGCTCGGCTGGCGAC	-3'
